# Carbon solubility and liquid–liquid immiscibility in Fe–C–S ternary system up to 6 GPa: implications for the core of small planetary bodies

**DOI:** 10.1186/s40645-026-00846-3

**Published:** 2026-09-20

**Authors:** Bin Zhao, Guillaume Morard, Geeth Manthilake, Paraskevas Parisiades, Jing Yang, Yingwei Fei, Yann Le Godec, Nozomi Kondo, Takashi Yoshino, Daniele Antonangeli

**Affiliations:** 1 UMR CNRS 7590, Institut de Minéralogie, de Physique des Matériaux et de Cosmochimie, IMPMC, Muséum National d’Histoire Naturelle, Sorbonne Université, 75005 Paris, France; 2 Institute for Planetary Materials, Okayama University, Misasa, Tottori, 682-0193 Japan; 3 Laboratoire Magmas et Volcans, Université Clermont-Auvergne, Clermont-Ferrand, 63000 France; 4 Earth and Planets Laboratory, Carnegie Institution for Science, 5251 Broad Branch Road, N.W., Washington, DC 20015 USA

**Keywords:** Iron–carbon–sulfur liquids, Miscibility, High pressure and high temperature, Planetary cores

## Abstract

High-pressure, high-temperature experiments were conducted in a multi-anvil press using seven different starting compositions to investigate the phase relations of Fe–C–S liquids at pressures up to 6 GPa and temperatures up to 2000 K. Quenched samples revealed immiscible C-rich and S-rich metallic liquids at 2 and 4 GPa, manifesting as either large immiscible zones or emulsified smaller droplets. On the contrary, no immiscibility was observed at 5 or 6 GPa. At these higher pressures, the carbon solubility limit, significantly reduced by increasing sulfur content in the ternary liquid, prevents the formation of a liquid phase rich in both C and S. Our findings thus indicate that super-liquidus immiscibility in Fe-rich compositions does not occur at pressures above 5 GPa, due to the differential pressure dependence of C solubility and miscibility. The limited C solubility in S-rich liquids at higher pressures effectively suppresses immiscibility. Based on these results, terrestrial planetary bodies with fluid cores at pressures exceeding 5 GPa are unlikely to experience liquid–liquid immiscibility, even when both carbon and sulfur are abundant, as sulfur limits carbon solubility and promotes its exsolution. For the Moon, immiscibility-induced core stratification is not expected, though local emulsification could occur near the core–mantle boundary (CMB). In smaller bodies, a two-liquid Fe–C–S core may form at high temperatures, but progressive cooling may lead to graphite crystallization and the potential development of a graphitic crust.

**Supplementary Information:**

The online version contains supplementary material available at https://doi.org/10.1186/s40645-026-00846-3.

**Figure:**
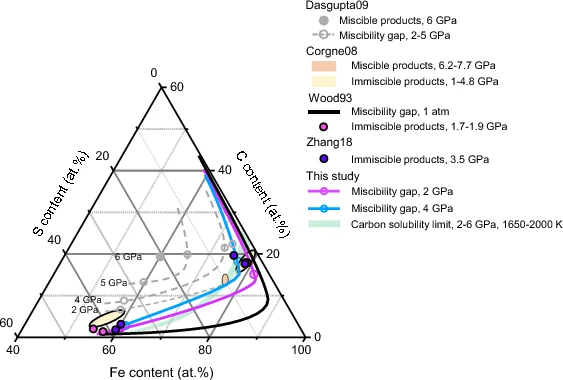

## Introduction

Proper knowledge of the structural and dynamical properties of planetary cores is fundamental to understand their formation and evolution. Solid vs. liquid nature, phase stability, chemical composition, and homogeneity are examples of key characteristics that can be directly related to the phase diagram of constituent materials under relevant conditions of pressure (P) and temperature (T). An at least partially molten, iron-rich core is widespread among telluric planets, as established for the Earth (e.g., Dziewonski and Anderson 1981; Irving et al. 2018), Mercury (Margot et al. 2007), Venus (Konopliv and Yoder 1996), and Mars (Yoder et al. 2003; Stähler et al. 2021; Le Maistre et al. 2023), but as well for smaller bodies such as the Moon (e.g., Garcia et al. 2011 and 2019; Viswanathan et al. 2019; Briaud et al. 2023). Besides the more common liquid–solid boundary, liquid–liquid immiscibility has been speculated to occur upon cooling, depending on the concentration of light elements and the specific P–T conditions. This phenomenon is particularly relevant in the metallic cores of small terrestrial bodies (e.g., Corgne et al. 2008; Morard and Katsura 2010). Immiscibility-induced core stratification can have far-reaching implications, not only for the planetary structural models, but also for global dynamics. These include the onset and sustainability of core convection and potentially dynamo generation, which directly influence the planetary magnetic field.

Sulfur (S) and carbon (C) are two light elements entering the composition of metallic cores of terrestrial planets and moons. Their presence is often advocated on the basis of the density reduction with respect to pure iron (e.g., Morard et al. 2013, 2018; Zhao et al. 2023), effects on sound velocities (e.g., Jing et al. 2014; Antonangeli et al. 2015; Nakajima et al. 2015; Nishida et al. 2016), and melting curves (Lord et al. 2009; Morard et al. 2011, 2017a). Under the P–T conditions relevant to small planetary bodies, sulfur and carbon have been proved to partition effectively into the iron core from chondrite-like starting compositions. Fe alloys enriched in C and S may exhibit immiscibility above the liquidus due to the mutual repulsion between C-rich and S-rich phases (Tafwidli and Kang 2017). This miscibility gap narrows with increasing pressure (Corgne et al. 2008; Dasgupta et al. 2009). Notably, carbon solubility in binary Fe–C liquids can reach up to 6–8 wt% (Wood 1993; Nakajima et al. 2009; Fei and Brosh 2014). However, when both C and S are present, their solubilities compete (Zhang et al. 2013; Tsuno et al. 2018). Despite extensive experimental and theoretical efforts to investigate C–S interactions in metallic melts under planetary core conditions (e.g., Wang et al. 1991; Corgne et al. 2008; Deng et al. 2013; Tsuno et al. 2018), discrepancies persist regarding carbon solubility, the evolution of the miscibility gap in the Fe–C–S system, and the effect of nickel (Ni) on C solubility. For instance, Dasgupta et al. (2009) reported both miscible and immiscible Fe–C–S liquids simultaneously enriched in C and S—an observation not reproduced by subsequent studies under similar P–T conditions. Zhang et al. (2018) found that Ni markedly reduces C solubility in Fe–C–S liquids, particularly in Fe-rich systems, whereas Tsuno et al. (2018) did not observe a comparable effect.

A likely source of these discrepancies may stem from the limited range of starting compositions explored in previous studies (Corgne et al. 2008; Dasgupta et al. 2009), particularly regarding the miscibility gaps in the Fe–C–S alloys. Additionally, the quantification of carbon solubility in S-rich liquids, and its influence on the evolution of the miscibility gap with increasing pressure, has not been thoroughly addressed.

The present study thus aims to precisely characterize the miscibility gap in the Fe–C–S system through a systematic investigation of a broad range of starting compositions within the Fe-rich corner of the ternary diagram, under P–T conditions relevant to the core of small telluric bodies. Specifically, we present results from quench experiments conducted using a multi-anvil press at pressures from 2 to 6 GPa and selected temperatures of 1650 K and 2000 K. Based on the compositional and textural analysis of the recovered samples, we discuss the evolution of the Fe–C–S liquid–liquid miscibility gap with pressure and the solubility of carbon in the liquid phase.

## Methods

### Sample preparation

The starting materials consisted in mixtures of iron (99.5%, Alfa Aesar), FeS (99.98, Alfa Aesar), and graphite powders (99%, Alfa Aesar). The powders were mixed with the nominal compositions: Fe_81_C_6_S_13_, Fe_78_C_8_S_14_, Fe_75_C_10_S_15_, Fe_70_C_12_S_18_, Fe_65_C_14_S_21_, Fe_58_C_20_S_22_, and Fe_44_C_25_S_31_ (compositions in at%, hereafter referred to as Fe#81, Fe#78, Fe#75, Fe#70, Fe#65, Fe#58, and Fe#44). Each mixture was ground in an agate mortar for 30 min to reduce the grain size and to achieve high homogeneity. Mixed powders were stored in glass tubes, which were placed in a vacuum box. Before loading into capsules, the powders were heated in a vacuum furnace at 150 ℃ for a few hours to ensure dehydration.

### Experiments at high pressure and high temperature

Tests experiments were carried out in a multi-anvil press with 18/11 assemblies at Laboratoire Magmas et Volcans (LMV), Université Clermont Auvergne, using hBN capsules to contain the samples (Figure S1). Analysis of the samples recovered from experiments quenched at 2 GPa and 1650 K revealed severe leakage, indicating that hBN may not be suitable for sealing iron-rich melts under these conditions. Therefore, sapphire or sintered alumina capsules were used for all subsequent experiments conducted either in a Walker-type multi-anvil press at Institut de Minéralogie, de Physique des Matériaux et de Cosmochimie (IMPMC), Sorbonne Université, or in a Kawai-type multi-anvil press at Institute for Planetary Materials (IPM), Okayama University. Cell designs are shown in Figure S2. Pressure was calibrated with the phase transition of quartz–coesite and fayalite–spinel in both institutes, and the uncertainty in pressure is estimated as less than 0.5 GPa. For 18/11 cell assemblies used at IPM, a dedicated run indicated the temperature uncertainty is about ± 60 K at 1800 K across the sample size (Zhao et al. 2026). In the case of 14/8 assemblies used at IMPMC, the thermocouples placed off-center are expected to result in a lower reading temperature of ~100 K (Leinenweber et al. 2012), which can be considered negligible given that both carbon solubility in liquid Fe and the miscibility gap of Fe–C–S alloy only moderately vary with temperature at close *P*–*T* conditions (Nakajima et al. 2009; Corgne et al. 2008).

To investigate the pressure dependence of the miscibility gap, experiments were first conducted at the lowest pressure of 2 GPa, starting from the most iron-rich starting compositions. The samples were heated with the current passing through the graphite heater, powered by a DC (at IMPMC) or AC (at IPM) power supply, and the temperature was monitored by a type D thermocouple (W-3Re/W-25Re). The cell was maintained at the target temperature for 1 h to ensure equilibrium, after which it was quenched by cutting the power supply. Due to the active cooling, the temperature dropped below 1000 K within 1 s and below 400 K within about 5 s after power shutdown.

The recovered samples were mounted in epoxy resin and polished for analysis. Examination of the quenched samples, obtained from experiments using compositions with progressively higher light element content, allowed the miscibility gap to be defined at 2 GPa. Subsequent experiments were carried out at progressively higher pressures (4, 5 and 6 GPa) using starting composition increasingly enriched in light elements. Following this protocol, the pressure dependence of Fe–C–S miscibility was constrained at both 1650 K and 2000 K.

### Analysis of the recovered samples

The microstructure and phases were analyzed using a Zeiss-55 scanning electron microscope (SEM) and energy-dispersive spectroscopy (EDS) mapping at IMPMC, Sorbonne University. Chemical composition analysis was performed using two different electron microprobes. At the Earth and Planets Laboratory, Carnegie Institution for Science, a JEOL8530F electron microprobe was operated at an accelerating voltage of 15 kV and a beam current of 30 nA. Samples were iridium-coated, and pure Fe, pyrite, and magnetite were used as calibrants for Fe, S, and O, respectively. At the Institute for Planetary Materials, Okayama University, a JEOL JXA-IHP200F field-emission electron microprobe was used at 15 kV with a beam current of 10 nA for Fe, O, and S (carbon-coated samples) and 100 nA for C (gold-coated samples). Calibrants used were pure Fe, pyrite, and hematite for Fe, S and O, respectively. Carbon quantification followed the protocol detailed in Dasgupta and Walker (2008) and Yang et al. (2019). For measurements at the Earth and Planets Laboratory, the calibration curve for carbon was established based on the C *Kα* intensity of Fe_7_C_3_, Fe_3_C, a C-bearing NIST steel of Fe−1 wt%C, and pure Fe, while at the Institute for Planetary Materials, the calibration was based on synthesized Fe_7_C_3_, Fe−0.295 wt%C and Fe−1.44 wt%C (manufactured by The Japan Iron and Steel Federation), and pure Fe. The calibration curves are shown in Figure S3.

During EPMA analysis at IPM, very low C counts were recorded in S-rich phases. Specifically, the S-rich phases in samples Fe#75 and Fe#44, analyzed at the C peak, exhibited a count rate ~300 below that of the Fe standard (~ 3700 versus 3996, Figure S3). Consequently, the carbon content of these phases was deemed below the detection limit and normalized to 0 (Table 1 and S1). Similar low C count rates for S-rich phases have been reported by Zhang et al. (2018), who attributed this phenomenon to variations in the thermal conductivity of alloys with differing S content. Given that C contamination stems from the thermally activated evaporation of C-H organics, such as grease and oil, the measured C content in S-rich samples tends to be systematically lower than the actual value. Our EPMA results show a total of overall 98–100% despite a few outliers, without a systematically deviation for S-rich samples. Normalizing C content below the detection limit to zero is thus considered to have no significant impact on the interpretation of the results or the subsequent discussion.

**Table 1:** Run products and normalized composition of the observed phases

Starting composition	Designation^a^	Pressure	Phase assemblage	Atomic percent^c^				Notes
				Fe	C	S	Total	
	*Runs at 1650 K*							
Fe_84_C_10_S_6_	Fe#84 (IMPMC)	2 GPa	Miscible liquid	84.7	9.1	6.2	100	Bulk composition
Fe_81_C_6_S_13_	Fe#81 (IMPMC)	2 GPa	Miscible liquid	77.3	8.3	14.4	100	Bulk composition
Fe_75_C_10_S_15_	Fe#75 (IPM)	2 GPa	Immiscible liquids	84.1	12.9	3.0	100	C-rich
				59.4	0	40.6	100	S-rich
Fe_70_C_12_S_18_	Fe#70 (IPM)	2 GPa	Immiscible liquids	80.2	17.5	2.3	100	C-rich
				56.7	1.9	41.4	100	S-rich
Fe_65_C_14_S_21_	Fe#65 (IMPMC)	2 GPa	Immiscible liquids + graphite	80.6	14.6	4.8	100	C-rich
				64.2	2.0	33.8	100	S-rich
Fe_70_C_12_S_18_	Fe#70 (IMPMC)	4 GPa	Miscible liquid + graphite	69.8	7.8	22.4	100	Bulk composition
Fe_65_C_14_S_21_	Fe#65 (IPM)	4 GPa	Miscible liquid + graphite	66.2	4.3	29.5	100	Bulk composition
Fe_58_C_20_S_22_^b^	Fe#58 (IPM)	4 GPa	Immiscible liquids + graphite	75.4	20.0	4.6	100	C-rich
				62.1	1.3	36.6	100	S-rich
Fe_65_C_14_S_21_	Fe#65 (IPM)	5 GPa	Miscible liquid + graphite	69.5	5.7	24.8	100	Bulk composition
Fe_58_C_20_S_22_	Fe#58 (IPM)	5 GPa	Miscible liquid + graphite	67.4	4.4	28.2	100	Bulk composition
	*Runs at 2000 K*							
Fe_84_C_10_S_6_	Fe#84 (IMPMC)	2 GPa	Miscible	82.6	9.7	7.7	100	Bulk composition
Fe_81_C_6_S_13_	Fe#81 (IMPMC)	2 GPa	Miscible	78.4	5.0	16.6	100	Bulk composition
Fe_75_C_10_S_15_	Fe#75 (IMPMC)	2 GPa	Immiscible	81.7	15.1	3.2	100	C-rich
				60.4	2.3	37.3	100	S-rich
Fe_75_C_10_S_15_	Fe#75 (IMPMC)	4 GPa	Miscible	71.4	8.2	20.4	100	Bulk composition
Fe_65_C_14_S_21_	Fe#65 (IPM)	4 GPa	Miscible liquid + graphite	65.8	3.7	30.5	100	Bulk composition
Fe_58_C_20_S_22_^b^	Fe#58 (IPM)	4 GPa	Immiscible liquids + graphite	76.8	18.2	5.0	100	C-rich
				61.1	2.8	36.1	100	S-rich
Fe_65_C_14_S_21_	Fe#65 (IPM)	5 GPa	Miscible liquid + graphite	70.2	5.5	24.3	100	Bulk composition
Fe_58_C_20_S_22_	Fe#58 (IPM)	5 GPa	Miscible liquid + graphite	68.8	2.9	28.3	100	Bulk composition
Fe_58_C_20_S_22_	Fe#58 (IMPMC)	6 GPa	Miscible liquid + graphite	69.2	3.6	27.2	100	Bulk composition
Fe_44_C_25_S_31_	Fe#44 (IMPMC)	6 GPa	Miscible liquid + graphite	60.4	0	39.6	100	Bulk composition

^a^In parenthesis, the site where the experiment was conducted is specified. Temperature uncertainty is ±60 K for runs at IPM and ±100 K for runs at IMPMC. See text for more details

^b^Samples recovered from multi-anvil experiments showing an emulsified texture comprising C-rich droplets ranging in size from 5 to 150 microns

^c^Normalized atomic composition ignoring the trace amount of oxygen in the sample. The carbon content below the detection limit is normalized to 0. Refer to Table S1 for the original EPMA data. Measurements were carried out in such a way as to avoid areas with holes, which are interpreted as detached graphite

## Results

### Phase relations and chemical compositions

**Fig. 1:**
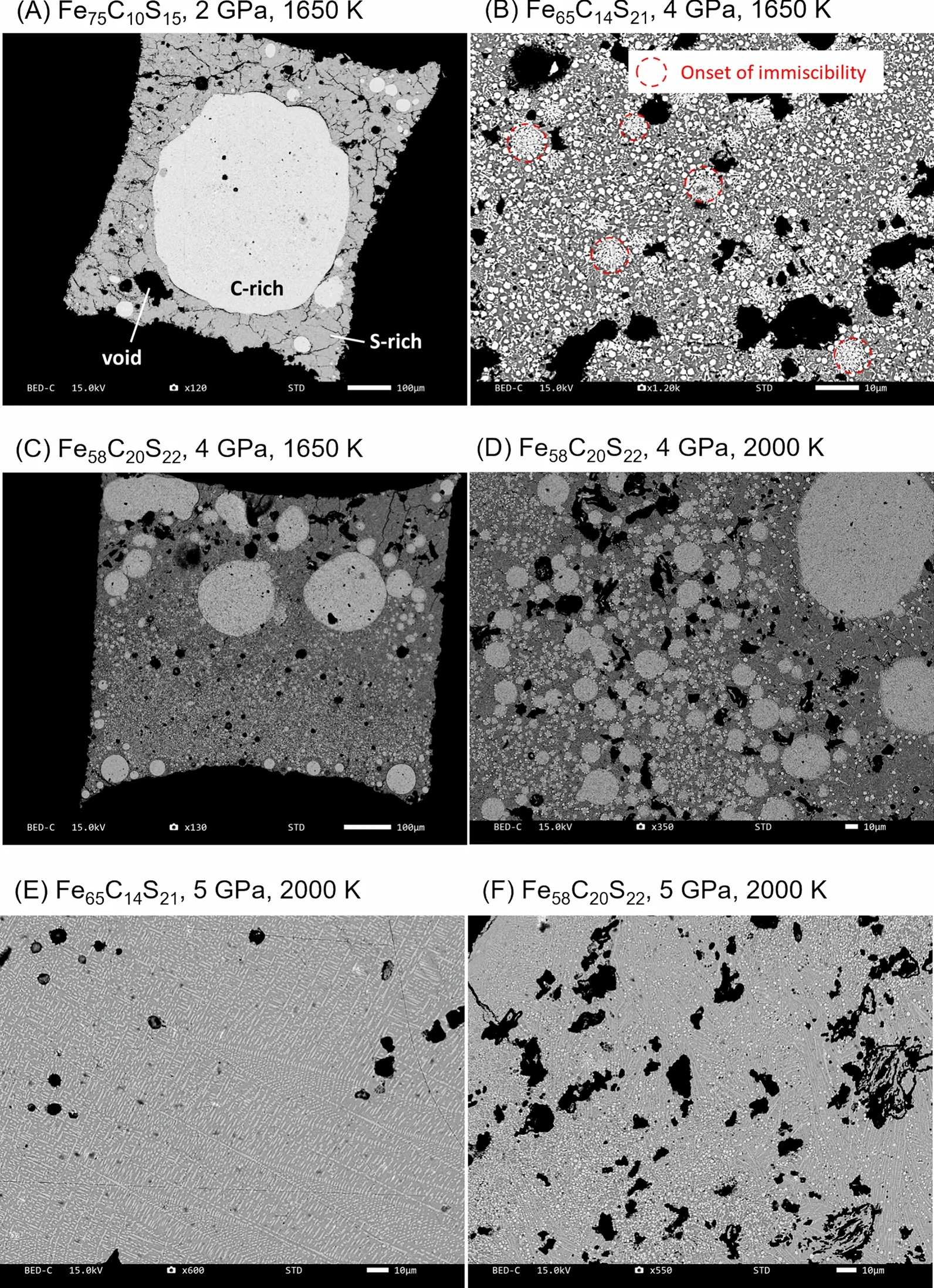
BSE images representative of textures in the recovered samples. Starting compositions and experimental conditions are marked on top of each image. **A**: Fully stratified immiscible texture with a large C-rich melt pool surrounded by S-rich liquid. **B**: Onset of immiscibility. C-rich liquid starts to gather and form droplets indicative of the bulk composition close to the miscibility gap. **C** and **D**: Immiscible sample with partly emulsified texture. Compared to the typical immiscible texture (**A**), the interfacial tension is not large enough to effectively separate the two liquids of close density. Dark areas are voids associated with detached graphite crystals during polishing. **E** and **F**: Homogeneous texture quenched from single liquid

Examples of back-scattered electron (BSE) images of samples with textures representative of quench from a single liquid and from two immiscible liquids are shown in Fig. 1. Experimental conditions and normalized chemical compositions of the recovered phases are summarized in Table 1 (compositions in atomic percent) and in Table S1 (compositions in weight percent). The quench textures characteristic of Fe–S or Fe–C liquids are clearly observed. Samples with simultaneously low C and S contents exhibit typical homogeneous, dendritic textures (Fig. 1A), where brighter regions are S-poor and darker regions are S-rich. Conversely, heterogeneous textures can be interpreted on the basis of the liquid/liquid immiscibility and C saturation in the melt, which are sometimes closely intertwined.

Immiscibility between C-rich and S-rich metallic liquids is observed when the Fe content in the starting material is 75 at% or less at 2 GPa, and 58 at% or less at 4 GPa, with no clear dependence on temperature. The textures are characterized either by large C-rich blobs (Fig. 1B) or by smaller C-rich droplets, ranging in size from 5 to 150 microns, surrounded by S-rich liquid (Fig. 1C and D). Since all examples shown in Fig. 1 are from experiments conducted at IPM and quenched using the same procedure, the variation in droplet size is unlikely to result from differences in quenching. A similar emulsification texture has also been reported for the Fe–Si–S system at 4 GPa (Morard and Katsura 2010). Terasaki et al. (2009 and 2012) pointed out that the size of metallic droplets in silicate melt is highly dependent on interfacial tension, which is influenced by pressure, temperature and composition. As pressure increases, the densities of C-rich and S-rich liquids converge. This may result in similar chemical bonding within the two liquids, reducing interfacial tension and preventing clear segregation. The observation that the size of C-rich droplets in samples quenched from Fe–C–S liquids is more sensitive to changes in starting composition and pressure than to temperature can be attributed to the comparatively smaller effect of temperature on the density difference between C-rich and S-rich liquids (Morard et al. 2017b; Xu et al. 2021; Zhao et al. 2023).

**Fig. 2:**
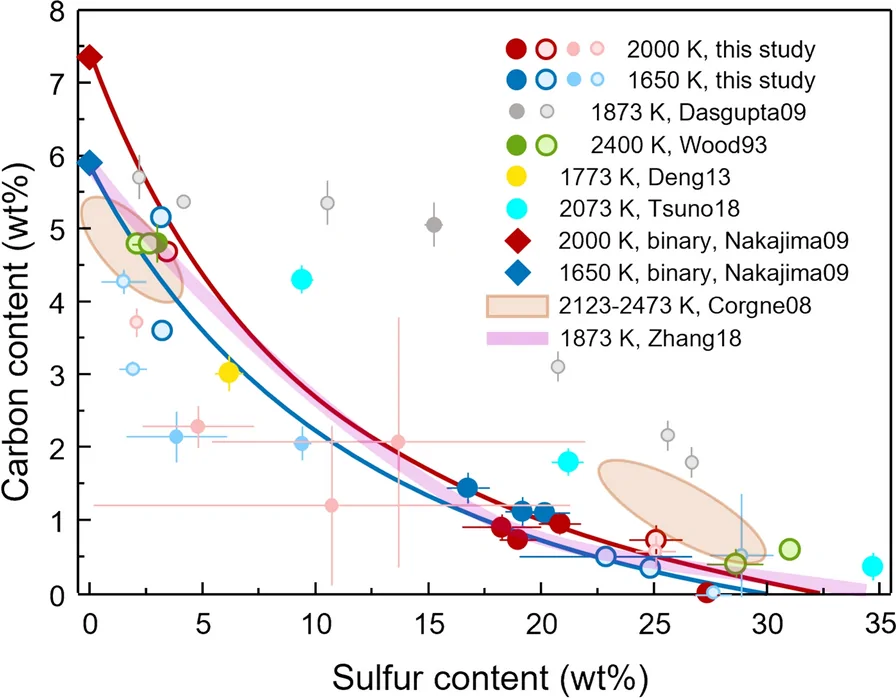
Carbon solubility limit in ternary Fe–C–S liquids in the 2–6 GPa range at 1650 K (blue line and symbols) and 2000 K (red line and symbols) as determined by the composition of C-saturated samples. Diamonds represent C-saturated composition in binary Fe–C liquid at 5 GPa (Nakajima et al. 2009). Together shown are results at similar P–T conditions from previous studies (Wood 1993; Corgne et al. 2008; Dasgupta et al. 2009; Deng et al. 2013; Tsuno et al. 2018; Zhang et al. 2018). Large symbols represent C-saturated compositions while small symbols represent C-undersaturated compositions from this study and literature. Miscibility and immiscibility are indicated by filled and open symbols, respectively

**Fig. 3:**
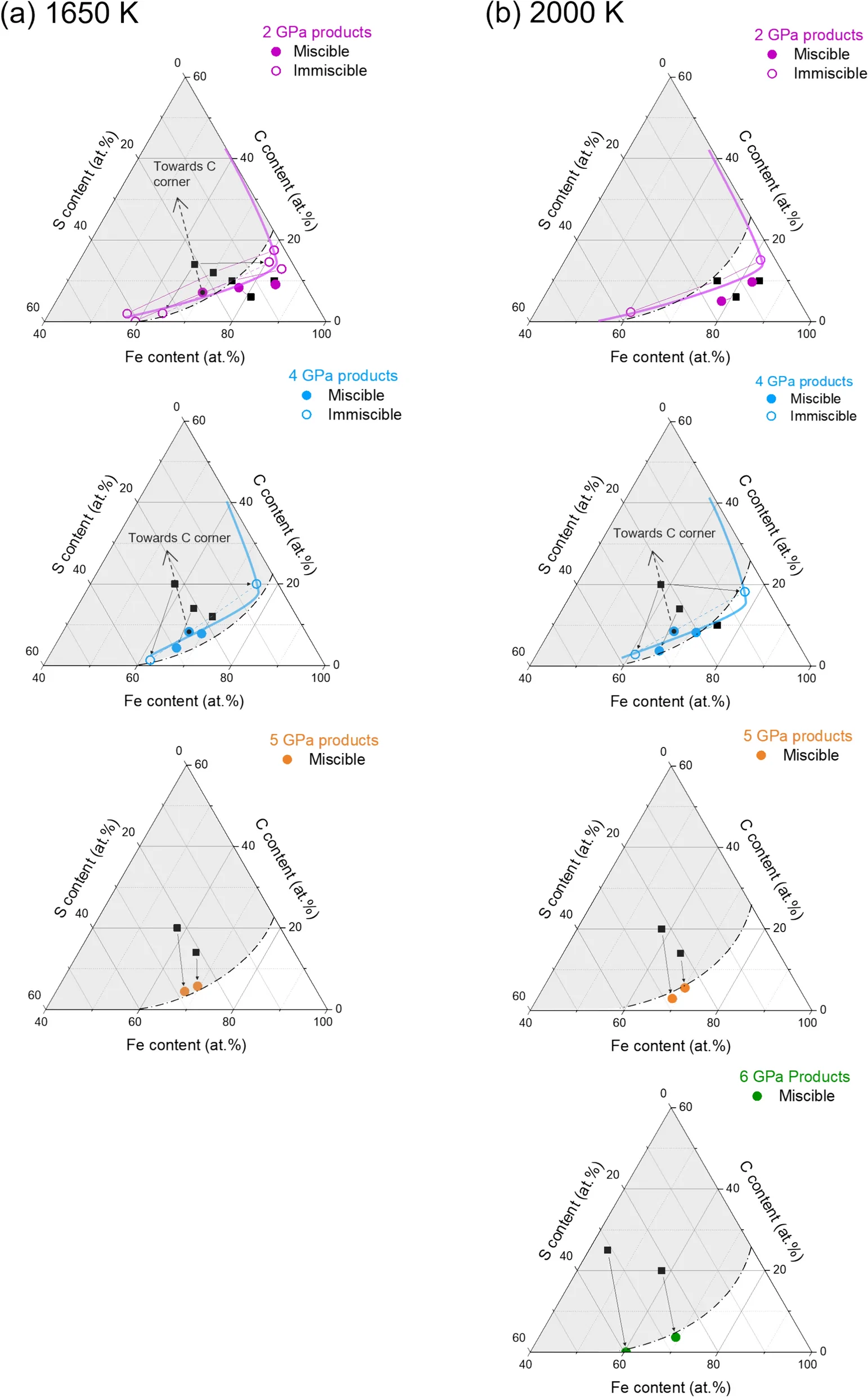
Starting composition (black solid squares), products (colored, empty and filled dots), and miscibility gaps (colored lines) of Fe–C–S alloys at 1650 K (left) and 2000 K (right) determined by the composition of recovered samples (color-coded for pressure dependence). Shaded areas delimited by the dash–dotted boundaries represent “*forbidden compositions*” for Fe–C–S liquid due to C saturation (Red line for 2000 K and blue line for 1650 K in Fig. 2). Under conditions of C undersaturation (Fe#84–Fe#70), the products are connected to starting composition by solid lines. In the cases where two liquids coexist with graphite (Fe#65–Fe#44), solid arrows link the starting composition to the products. For immiscible samples at 2 GPa, the starting composition roughly lies on the tie line of the immiscible products. Double-filled points indicate the “effective” starting composition for the C oversaturated samples, inferred by the intersection of the line connecting the two immiscible products and a line running from the C corner through the nominal starting composition (dashed arrows). The miscibility gap at 2 and 4 GPa was determined from the composition of all the products. No significant difference is observed between results at 1650 K and 2000 K

Carbon saturation is revealed by the presence of graphite grains within the melt, resulting in black areas in Fig. 1C–F. Distinguishing signatures of graphite grains from other polishing-induced holes can be challenging, as fragile graphite grains are prone to detachment during polishing. Noteworthy, carbon saturation occurs in all the samples with more than 12 at% C in the starting composition (Fig. 2). This notion is further supported by the observation that, for these C-rich samples, extrapolating the lines connecting the products to the starting composition roughly points toward the C corner of the ternary diagram (Fig. 3), as discussed in more detail later.

### Carbon solubility in liquid Fe–C–S alloys

On the C-rich side of binary Fe–C liquid, graphite or diamond, depending on the pressure, exsolves as the solid phase below liquidus (Nakajima et al. 2009; Fei and Brosh 2014). The carbon content of the coexisting liquid defines the carbon solubility limit at given P–T conditions. The solubility limit of C in a binary Fe–C liquid is reported to be 6–8 wt% (Nakajima et al. 2009), depending only marginally on temperature (see Fig. 2) and not significantly on pressure.

Following a similar protocol, quantitative analysis of the C content of our quenched samples, and in particular of liquid coexisting with exsolved graphite, allowed us to define the carbon solubility limit for the ternary Fe–C–S system in the 2–6 GPa range (Fig. 2).

The compositions of all recovered samples are presented in Fig. 2, along with binary Fe–C data from Nakajima et al. (2009) and Fe–C–S data from the literature. Regardless of the starting composition, C saturation, or the miscible/immiscible nature of the liquids, the carbon content of the recovered liquids defines a clear trend. This consistency supports the reliability of our solubility estimates, which are in agreement with most of the previous studies, with the exception of Dasgupta et al. 2009. As previously noted, similar to Zhang et al. (2018), we observed very low C counts in S-rich phases. The difference in determined C solubility between the results of Zhang et al. (2018) and this study is negligible, despite minor discrepancies at the S-rich end (Fig. 2). A detailed discussion is provided in Sect. 4. Compared to binary Fe–C liquid, carbon solubility in ternary Fe–C–S liquids is lower and decreases with increasing sulfur content.

### Miscibility of the Fe–C–S alloys

Figure 3 shows the starting compositions and experimental products plotted on a ternary diagram at 2, 4, and 5 GPa at 1650 K, and at 2, 4, 5, and 6 GPa at 2000 K. Shaded areas delimited by the dash-dotted boundaries represent compositions beyond C solubility limit. The products are related to the corresponding starting composition by solid lines when undersaturated in C, while solid arrows link the starting composition to the products in the case of C oversaturation.

Figure 3 shows that, for the compositions richest in light elements (Fe#65 and below), both the miscible and immiscible products diverge significantly from the starting composition toward C-poor side. For the miscible products at 5 and 6 GPa (orange and green filled points), the reverse extension of the arrows roughly points to the C corner, indicating that the liquid is only C-deficient compared to the starting composition. Conversely, at 4 GPa (blue filled points), the miscible product is slightly poorer in Fe other than in C, probably due to a small amount of Fe_3_C crystallized and attached to graphite grains during quenching, and detached with the graphite during polishing. For immiscible samples, the starting composition does no longer lie on the tie line of the immiscible products for Fe#65 and below. In all these cases, plenty of pores of irregular shapes are observed on the surface of the recovered samples (Fig. 1, C, E, and F). Similar dark regions have also been observed before on the polished surface of Fe–C binary samples (Nakajima et al. 2009) and interpreted as in the present case as the signature of graphite formation. However, unlike Nakajima et al. (2009), we consider these graphite grains to be exsolution products coexisting with the metallic melts rather than being formed during quenching, as their size is much larger than the texture scale (~ 1 μm). These metallic melts should therefore be oversaturated in C under experimental conditions. In these cases, the “effective” starting composition (double-filled points in Fig. 3) leading to the formation of the immiscible products can be determined by the intersection of the line connecting the two immiscible products with the line running from the C corner of the ternary diagram through the nominal starting composition (dashed arrow in Fig. 3).

Experiments were performed at 1650 K and 2000 K to study the effect of temperature on the miscibility gap. 1650 K is about the lower limit of the temperature range at which all samples are fully molten according to the previously reported Fe–C–S melting experiments (Dasgupta et al. 2009; Deng et al. 2013), while 2000 K is about the maximum temperature the cell can reach. It should also be noted that this temperature range covers the temperatures expected in the cores of small terrestrial planetary bodies (e.g., the Moon as from Wieczorek et al. (2006)). Miscibility gaps estimated at 1650 K and 2000 K on the basis of the composition of miscible products and the phase-sorted composition of immiscible products are shown in Fig. 3, color-coded for pressure dependence. Immiscible liquids were found at 2 and 4 GPa, and no noticeable temperature dependence was shown between 1650 and 2000 K. As the miscibility gap narrows effectively with increasing pressure while the carbon solubility limit does not substantially evolve with pressure, the very limited carbon content in S-rich liquids at higher pressure directly precluded the occurrence of immiscibility above 5 GPa.

## Discussion

### Comparison with previous studies

Comparison of the carbon solubility limit in Fe–S liquids determined in the present work with previous studies (Corgne et al. 2008; Tsuno et al. 2018) indicates that carbon solubility is only slightly dependent on temperature (Fig. 3), especially in S-rich liquids. Our results are also largely consistent with those of Zhang et al. (2018), the pink line in Fig. 2. The main uncertainty in precisely defining the miscibility line arises from the quench textures, which lead to some scatter in the data. In few cases, the composition exceeds the defined C solubility limit, as observed both in this study and in Zhang et al. (2018). Most importantly, we did not reproduce any liquid, whether miscible or immiscible, simultaneously enriched in both C and S, as reported by Dasgupta et al. (2009). There is therefore a clear limit for simultaneous C and S presence in liquid planetary cores. Differences in oxygen fugacity, imposed by varying experimental assemblies, do not appear to be the source of this discrepancy. Indeed, our results and those of Zhang et al. (2018), which used alumina and graphite sleeves, are in overall agreement, while in both our experiments and in those of Dasgupta et al. (2009), the samples were in direct contact with MgO, with graphite heaters surrounding the capsule.

**Fig. 4:**
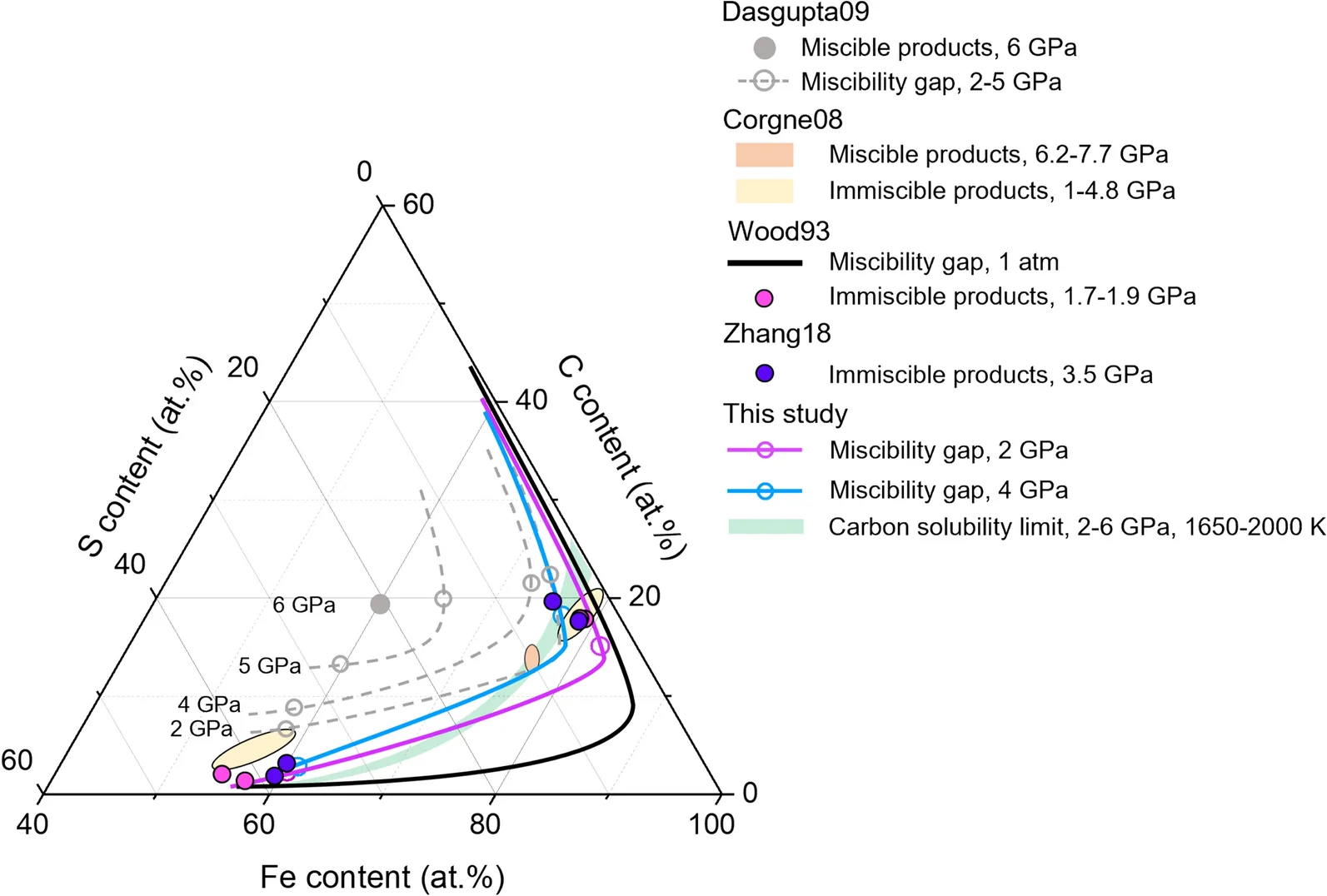
Comparison of the pressure evolution of the miscible/immiscible products and miscibility gap across different studies. Gray symbols and dashed lines are composition of run products and established miscibility gaps by Dasgupta et al. (2009). Filled dark blue and magenta symbols represent immiscible products from Zhang et al. (2018) and Wood (1993), respectively. For clarity, the miscibility gaps reported by Corgne et al. (2008) are not shown in the figure, as they essentially overlap with the results of this study. Instead, we present the compositions of the miscible and immiscible products as shaded areas

These discrepancies in the carbon solubility of the produced melt directly and inevitably result in a distinct miscibility gap and a markedly different pressure evolution. Since the effect of temperature on the miscibility gap between 1650 and 2000 K is overall minor (Fig. 3), our results at 2000 K are used for comparison in Fig. 4. The miscibility gaps we defined at 2 and 4 GPa are closer to those reported by Corgne et al. (2008) than to those by Dasgupta et al. (2009). A slight difference does exist between our study and that of Corgne et al. (2008) regarding the composition of S-rich phase, with the latter reporting a slightly higher S content. This difference could be attributed to increased C solubility in the S-rich phase at higher temperature, as the experiments conducted by Corgne et al. (2008) were performed within the 2123–2473 K range. Alternatively, it could be due the presence of Ni, which was absent in our experiments. While Corgne et al. (2008) suggested that oxygen in the liquid alloy can slightly increase carbon solubility, this is unlikely to be a source of inconsistency with our work, since the recovered samples show similar oxygen in both studies. On the other hand, the compositions of the immiscible products are consistent, within error bars, with those reported in most of the previous studies. Notably, the extremely low carbon concentration on S-rich side, regardless of pressure, suggests that the pressure-induced evolution of the miscibility gap may be more gradual than previously estimated. Furthermore, although both our results and those of Dasgupta et al. (2009) indicate no immiscibility above 5 GPa, the underlying mechanisms differ significantly. The C solubility limit, as defined by C-saturated samples in our experiments, effectively prevents the system from entering the miscibility gap at 5 GPa. In other words, above 4 GPa, liquids rich in light elements reach the C solubility threshold before immiscibility can occur (Fig. 4). Increasing temperature does not significantly affect the extent of immiscibility above 4 GPa, as temperature has only a minimal influence on carbon solubility.

### Implications for the cores of small planetary bodies

Recent experimental studies highlight the critical role of light elements in governing immiscibility in planetesimal cores during core formation (Bromiley et al. 2024). While terrestrial bodies may host volatile-rich cores, including carbon-saturated cores as hypothesized for Mercury (Xu et al. 2024), immiscibility is unlikely in the cores of medium-to-large bodies. Because immiscibility does not occur in Fe–C–S liquids above 5 GPa due to the limited carbon solubility in S-rich liquids, terrestrial planets with a core at pressures of 5 GPa or higher are not expected to experience liquid partitioning or immiscibility-induced core stratification at temperatures above 1650 K regardless of bulk C and S content. In contrast, pressure and composition emerge as the dominant controls on immiscibility in the Fe–C–S core of small bodies.

We note, however, that the Fe–C–S system has the lowest pressure threshold for the closure of the miscibility gap among core-forming ternary systems (Fe–S–X, where X = C, O, or Si). The miscibility gaps of Fe–S–O and Fe–S–Si systems close at much higher pressures (> 15 GPa; Tsuno et al. 2007; Sanloup and Fei 2004; Morard and Katsura 2010). If silicon or oxygen is present alongside carbon in liquid iron–sulfur alloys, the reported conclusions here might not strictly hold anymore, and the miscibility gap could extend to higher pressure. Most importantly, the interplay among multiple light elements likely increases the complexity of phase compositions at varying pressure–temperature conditions, calling for dedicated experiments.

The presence of nickel could also have an effect as it may suppress carbon solubility in Fe–Ni–C–S liquids at pressures of 2–5 GPa (Zhang et al. 2018), though the variability in their data suggests some uncertainty. Appreciable nickel content could thus stabilize miscible liquids up to 5 GPa, but its effect on carbon solubility weakens above 6 GPa (Tsuno et al. 2018).

#### Stratification in small Fe–C–S cores

For Fe–C–S cores, immiscibility is constrained by a pressure threshold of ~4–5 GPa. Below this threshold, Fe alloys enriched in carbon and sulfur can differentiate into immiscible C-rich and S-rich metallic liquids at high temperatures, particularly when minor elements like nickel and oxygen are present. Immiscibility also requires a bulk sulfur content constrained between 2 and 25 at%, as defined by the carbon solubility limit and the miscibility gap (see in 2- and 4-GPa diagrams). In carbon-oversaturated systems, the compositions of the C-rich and S-rich phases coexisting with graphite may shift slightly with cooling, as dictated by the Gibbs phase rule. As secular cooling progresses, the crystallization of a second solid phase, Fe₃C (Deng et al. 2013), will effectively suppress immiscibility by further limiting the degrees of freedom in the system. Specifically, a four-phase assemblage composed of Fe₃C + C + C-rich liquid + S-rich liquid would be highly unlikely to persist in planetary cores.

Limited carbon solubility in liquid Fe–S alloys can have major consequence, ranging from the formation of a diamond layer at the CMB, as envisaged for Mercury (Xu et al. 2024), to the more plausible development of a graphitic crust (e.g., Vander Kaaden et al. 2020; Steenstra and Westrenen 2020). Our results extend this possibility to small planetary bodies with core pressures below 4 GPa, provided they are sufficiently enriched in carbon and sulfur.

Specifically, considering the Moon with the core pressure ranging from ~4.6 at the CMB to 5.3 GPa at the center (Kuskov and Kronrod 1998; Garcia et al. 2011), both S and C efficiently partitioned into its core (Corgne et al. 2008; Steenstra and van Westrenen 2017; Steenstra et al. 2017), whereas O partitioning is very limited (Corgne et al. 2008). Si can also be considered incompatible in the Moon’s core due to the relatively high oxygen fugacity (Wadhwa 2008). To account for core densities inferred from seismic and/or geodetic studies (e.g., Garcia et al. 2019; Viswanathan et al. 2019; Kuskov et al. 2021), a substantial amount of light elements is required to explain the core density deficit relative to liquid iron. S content would need to exceed ~12 wt% in a fully molten core, while models with a solid inner core require at least ~5 wt% sulfur in the liquid outer core (Zhao et al. 2023). Carbon content in the liquid core would thus be effectively limited— below ~2 wt% in the former case and below ~4 wt% in the latter. Given these light element contents, miscibility-induced stratification is unlikely even with secular cooling, tough substantial emulsification could still occur if the lunar core is rich in both carbon and sulfur.

Future core seismic attenuation studies may reveal local emulsification or immiscibility, as seismic waves are sensitive to radial inhomogeneities (Stähler et al. 2021). Confirming such features would help constrain the species and content of elements alloyed with iron in the core.

#### Percolation and immiscibility

The process of core formation via liquid percolation is strongly influenced by liquid immiscibility, which introduces complexity into the differentiation of planetary bodies. Experimental and theoretical studies (e.g., Terasaki et al. 2008; Ghanbarzadeh et al. 2017) argue that immiscibility in Fe–C–S liquids can lead to the segregation of distinct metallic phases—Fe-rich (and potentially carbon-enriched) and FeS-rich (sulfur-enriched and carbon-poor)—each exhibiting unique percolation behaviors due to differences in density, viscosity, and wetting properties. In this context, the efficiency of drainage for each immiscible component and the composition of the resulting metallic core depend critically on wetting properties. Consequently, the core composition of a differentiated body may markedly differ depending on whether the core formed via percolation processes of a homogeneous liquid or of two distinct liquids.

In small planetary bodies, where internal pressures remain below 5 GPa, the Fe–C–S system exhibits a miscibility gap. Notably, C-rich liquids generally have metal–silicate dihedral angles well above the 60° connectivity threshold and are thus expected to form isolated droplets in a crystalline silicate matrix (Dong et al. 2019). Equilibrium percolation of such liquids under static conditions is therefore unlikely. In contrast, the coexisting S-rich liquid is expected to exhibit substantially better wetting properties, particularly at low pressure and under relatively oxidizing conditions, enabling it to form an interconnected network along silicate grain edges (Terasaki et al. 2008). Fe–C–S immiscibility may therefore drive selective segregation: The S-rich liquid drains first, while a portion of the C-rich, S-poor liquid remains trapped. Additionally, the higher melting point of C-rich liquid (e.g., Fei and Brosh 2014) may further impede carbon transport toward the core via solid-state diapirism.

Conversely, in larger planetary bodies with core pressures exceeding 5 GPa, the immiscibility gap closes, and core formation is dominated by the percolation of a homogeneous metallic liquid. In this scenario, increasing pressure progressively reduces percolation efficiency, as it increases the dihedral angle for a constant composition (Agee and Shannon 1999; Terasaki et al. 2008). Percolation-driven core formation is thus less likely in large bodies unless silicate partial melting or deformation, which could facilitate the percolative segregation, is involved (Hustoft and Kohlstedt 2006; Breg et al. 2017).

Ultimately, liquid immiscibility acts as a compositional filter during core formation, altering the distribution of light elements and shaping the long-term evolution of planetary interiors. The interplay between immiscibility, pressure, cooling rate, and physical properties of melts underscores the dynamic and multifaceted nature of planetary differentiation.

## Conclusions

Our study refines the understanding of pressure-induced closure of the miscibility gap in liquid Fe–S–C, highlighting the critical consequences of reduced C solubility in S-rich ternary liquids. In particular, our results demonstrate that super-liquidus Fe–C–S immiscibility does not occur above 5 GPa due to differential pressure dependence of C solubility and liquid miscibility. Below 4 GPa, the occurrence of immiscibility is highly dependent on the bulk S content, as determined by the intersectant of C solubility limits and the miscibility gap.

For terrestrial planetary bodies with Fe–C–S fluid cores at pressures exceeding 5 GPa, liquid–liquid immiscibility is unlikely to occur regardless of C and S content, since the presence of sulfur suppresses carbon dissolution in the core. This constraint may promote the formation of a graphitic crust and/or the stabilization of a diamond layer at the CMB, depending on the planet’s thermal history (Vander Kaaden and McCubbin 2015; Xu et al. 2024).

In the context of percolative core formation, the occurrence of liquid immiscibility would significantly influence the final core composition. It could lead to distinct core compositions even when derived from identical bulk starting materials, and potentially result in the retention of sulfur in the mantle and a corresponding depletion of sulfur in the core. For the Moon, immiscibility-induced core stratification is not expected; however, localized emulsification may occur near the CMB. In even smaller terrestrial bodies, a two-liquid Fe–C–S core could initially form at relatively high temperatures. As secular cooling progresses, the reduction in carbon solubility would eventually lead to the crystallization of graphite or C-rich phases.

## Supplementary Information

Additional file1 (DOCX 171 KB)

## Data Availability

The SEM images of all the samples are archived in the online repository Zenodo (Zhao 2025. https://doi.org/10.5281/zenodo.15052416).
