# Supplementary material for: Carbon solubility and liquid–liquid immiscibility in Fe–C–S ternary system up to 6 GPa: implications for the core of small planetary bodies
Source: Prog Earth Planet Sci. 2026 Sep 20;13(1):60. doi: 10.1186/s40645-026-00846-3 (PMC13590444; doi:10.1186/s40645-026-00846-3)
Supplement: Supplementary file 1 [file 40645_2026_846_MOESM1_ESM.docx]

**Supplementary information for ‘Carbon solubility and miscibility of Fe-C-S liquids up to 6 GPa: Implications for the core of planetary bodies’**

Bin Zhao^1,2*^, Guillaume Morard^1^, Geeth Manthilake^3^, Paraskevas Parisiades^1^, Jing Yang^4^, Yingwei Fei^4^, Yann Le Godec^1^, Nozomi Kondo^2^, Takashi Yoshino^2^, Daniele Antonangeli^1^

^1^Muséum National d’Histoire Naturelle, Sorbonne Université, UMR CNRS 7590, Institut de Minéralogie, de Physique des Matériaux et de Cosmochimie, IMPMC, 75005 Paris, France

^2^Institute for Planetary Materials, Okayama University, Misasa, Tottori 682-0193, Japan

^3^Laboratoire Magmas et Volcans, Université Clermont-Auvergne, Aubière, France

^4^Earth and Planets Laboratory, Carnegie Institution for Science, 5251 Broad Branch Road, N.W., Washington, DC 20015, USA

*Corresponding author Email address: zhaobin027@gmail.com

Figure S1. 18/11 multi-anvil cell assembly used in test runs at LMV, Université Clermont Auvergne

Figure S2. Left: 14/8 multi-anvil cell assembly from COMPRESS company used in quench experiments at IMPMC, Sorbonne Université. Right: 18/11 multi-anvil cell assembly used in quench experiments at IPM, Okayama University.

Figure S3. Carbon count rate in the Fe and Fe-C standards. The linear fit is used as a calibration for the correction of carbon content in the samples. Left: EPL, Carnegie Institution for Science; Right: IPM, Okayama University. The red mark on the y axis of the right figure shows the observed lower count rate (~ 3700 CPS) in S-rich sample than that in the Fe standard.

Table S1. Run products and EPMA results of the miscibility experiments

| Starting composition | | Designation | Pressure | Miscibility | Weight percent | | | | | Notes |
| --- | --- | --- | --- | --- | --- | --- | --- | --- | --- | --- |
|  |  |  |  |  | Fe | C** | S | O | Total |  |
|  | 1650-K runs | | | | | | | | | |
| Fe_84_C_10_S_6_ | | Fe#84 | 2 GPa | Miscible | 92.00(175) | 2.14(35) | 3.85(224) | 0.09(6) | 98.08(124) | Bulk composition |
| Fe_81_C_6_S_13_ | | Fe#81 | 2 GPa | Miscible | 88.17(34) | 2.05(23) | 9.42(40) | 0.67(5) | 100.31(39) | Bulk composition |
| Fe_75_C_10_S_15_ | | Fe#75 | 2 GPa | Immiscible | 93.12(81) | 3.07(10) | 1.92(61) | 0.02(3) | 98.13(45) | C-rich |
|  |  |  |  |  | 70.47(93) | / | 27.63(80) | 0.10(13) | 98.20(60) | S-rich |
| Fe_70_C_12_S_18_ | | Fe#70 | 2 GPa | Immiscible | 91.20(94) | 4.27(17) | 1.50(100) | 0.17(7) | 97.04(49) | C-rich |
|  |  |  |  |  | 68.89(171) | 0.51(85) | 28.87(141) | 0.06(8) | 98.32(55) | S-rich |
| Fe_65_C_14_S_21_ | | Fe#65 | 2 GPa | Immiscible | 92.76(50) | 3.60(11) | 3.20(45) | 0.23(8) | 99.79(47) | C-rich |
|  |  |  |  |  | 75.55(278) | 0.50(10) | 22.85(382) | 0.40(18) | 99.30(144) | S-rich |
| Fe_70_C_12_S_18_ | | Fe#70 | 4 GPa | Miscible | 83.74(56) | 2.02(25) | 15.42(65) | 0.98(21) | 102.16(33) | Bulk composition |
| Fe_65_C_14_S_21_ | | Fe#65 | 4 GPa | Miscible | 77.9(101) | 1.10(12) | 19.91(110) | 0.26(5) | 99.17(40) | Bulk composition |
| Fe_58_C_20_S_22_* | | Fe#58 | 4 GPa | Immiscible | 90.50(69) | 5.16(12) | 3.16(39) | 0.05(4) | 98.87(51) | C-rich |
|  |  |  |  |  | 73.41(70) | 0.34(9) | 24.83(43) | 0.10(10) | 98.68(39) | S-rich |
| Fe_65_C_14_S_21_ | | Fe#65 | 5 GPa | Miscible | 80.05(112) | 1.41(21) | 16.40(95) | 0.15(7) | 98.01(48) | Bulk composition |
| Fe_58_C_20_S_22_ | | Fe#58 | 5 GPa | Miscible | 78.29(105) | 1.09(20) | 18.82(76) | 0.26(11) | 98.46(80) | Bulk composition |
|  | 2000-K runs | | | | | | | | | |
| Fe_84_C_10_S_6_ | | Fe#84 | 2 GPa | Miscible | 90.21(264) | 2.28(29) | 4.80(248) | 0.05(5) | 97.34(87) | Bulk composition |
| Fe_81_C_6_S_13_ | | Fe#81 | 2 GPa | Miscible | 88.16(962) | 1.20(110) | 10.72(1056) | 0.45(17) | 100.53(80) | Bulk composition |
| Fe_75_C_10_S_15_ | | Fe#75 | 2 GPa | Immiscible | 93.25(39) | 3.71(20) | 2.08(6) | 0.39(6) | 99.43(47) | C-rich |
|  |  |  |  |  | 70.70(54) | 0.57(21) | 25.07(89) | 1.44(25) | 97.78(24) | S-rich |
| Fe_75_C_10_S_15_ | | Fe#75 | 4 GPa | Miscible | 83.30(695) | 2.07(172) | 13.68(829) | 0.69(034) | 99.74(122) | Bulk composition |
| Fe_65_C_14_S_21_ | | Fe#65 | 4 GPa | Miscible | 77.83(95) | 0.94(15) | 20.72(93) | 0.20(5) | 99.69(68) | Bulk composition |
| Fe_58_C_20_S_22_* | | Fe#58 | 4 GPa | Immiscible | 91.68(59) | 4.68(11) | 3.42(23) | 0.03(4) | 99.81(68) | C-rich |
|  |  |  |  |  | 73.94(73) | 0.73(20) | 25.07(119) | 0.25(14) | 99.99(62) | S-rich |
| Fe_65_C_14_S_21_ | | Fe#65 | 5 GPa | Miscible | 80.82(72) | 1.37(21) | 16.03(66) | 0.10(10) | 98.32(35) | Bulk composition |
| Fe_58_C_20_S_22_ | | Fe#58 | 5 GPa | Miscible | 78.19(100) | 0.70(5) | 18.50(83) | 0.25(9) | 97.64(64)) | Bulk composition |
| Fe_58_C_20_S_22_ | | Fe#58 | 6 GPa | Miscible | 78.84(94) | 0.89(18) | 17.79(174) | 0.46(10) | 97.98(90) | Bulk composition |
| Fe_44_C_25_S_31_ | | Fe#44 | 6 GPa | Miscible | 72.41(322) | / | 27.46(59) | 0.96(27) | 100.83(64) | Bulk composition |

* Recovered samples from multi-anvil experiments show an emulsified texture including C-rich droplets sized 5 – 150 microns.

** The measurements have avoided the hollow regions which are thought to be the detached C-rich phases (C-rich droplets, Fe_3_C grains and graphite grains). The slashes indicate that the carbon content is below the detection limit.
